# Semantic Incongruency Interferes With Endogenous Attention in Cross-Modal Integration of Semantically Congruent Objects

**DOI:** 10.3389/fnint.2019.00053

**Published:** 2019-09-11

**Authors:** Julia Spilcke-Liss, Jun Zhu, Sebastian Gluth, Michael Spezio, Jan Gläscher

**Affiliations:** ^1^Institute for Systems Neuroscience, University Medical Center Hamburg-Eppendorf, Hamburg, Germany; ^2^Department of Computer Science, Tsinghua University, Beijing, China; ^3^Department of Psychology, University of Basel, Basel, Switzerland; ^4^Psychology and Neuroscience, Scripps College, Claremont, CA, United States

**Keywords:** cross-modal integration, semantic congruency, exogenous attention, endogenous attention, drift diffusion model

## Abstract

Efficient multisensory integration is often influenced by other cognitive processes including, but not limited to, semantic congruency and focused endogenous attention. Semantic congruency can re-allocate processing resources to the location of a congruent stimulus, while attention can prioritize the integration of multi-sensory stimuli under focus. Here, we explore the robustness of this phenomenon in the context of three stimuli, two of which are in the focus of endogenous attention. Participants completed an endogenous attention task with a stimulus compound consisting of 3 different objects: (1) a visual object (V) in the foreground, (2) an auditory object (A), and (3) a visual background scene object (B). Three groups of participants focused their attention on either the visual object and auditory sound (Group VA, *n* = 30), the visual object and the background (VB, *n* = 27), or the auditory sound and the background (AB, *n* = 30), and judged the semantic congruency of the objects under focus. Congruency varied systematically across all 3 stimuli: All stimuli could be semantically incongruent (e.g., V, ambulance; A, church bell; and B, swimming-pool) or all could be congruent (e.g., V, lion; A, roar; and B, savannah), or two objects could be congruent with the remaining one incongruent to the other two (e.g., V, duck; A, quack; and B, phone booth). Participants exhibited a distinct pattern of errors: when participants attended two congruent objects (e.g., group VA: V, lion; A, roar), in the presence of an unattended, incongruent third object (e.g., B, bath room) they tended to make more errors than in any other stimulus combination. Drift diffusion modeling of the behavioral data revealed a significantly smaller drift rate in two-congruent-attended condition, indicating slower evidence accumulation, which was likely due to interference from the unattended, incongruent object. Interference with evidence accumulation occurred independently of which pair of objects was in the focus of attention, which suggests that the vulnerability of congruency judgments to incongruent unattended distractors is not affected by sensory modalities. A control analysis ruled out the simple explanation of a negative response bias. These findings implicate that our perceptual system is highly sensitive to semantic incongruencies even when they are not endogenously attended.

## Introduction

Cross-modal, multi-sensory integration is one of the most remarkable achievements of perceptual processing as it enables the binding of information from different sensory modalities into a single coherent percept [see, e.g., (Senkowski et al., 2008) for a review]. Yet the efficiency of integration is influenced by several modulating factors including, but not limited to, spatial and temporal proximity (Meredith and Stein, 1986a, b), and semantic congruency (Taylor et al., 2006; Doehrmann and Naumer, 2008; Steinweg and Mast, 2017). By varying these modulating factors and observing their effects on multi-sensory integration, we can study how the brain accomplishes the requisite binding processes, along with the role of endogenous attention. To capture these dynamics requires a design that engages endogenous attention in selecting at least two objects for comparison, in the presence of at least one distractor, and controlling for modality.

Several studies point to the notion that attention is likely critical for the advantage that semantic congruence confers upon cognitive processes of cross-modal integration. For instance, recent accounts demonstrate a performance advantage for semantically congruent multisensory stimuli during visual search (Iordanescu et al., 2008, 2010), but only under low cognitive load (Matusz et al., 2015). Furthermore, semantic congruency of multi-modal stimuli facilitates perceptual processing of unrelated material at the same location of the congruent multisensory prime (Mastroberardino et al., 2015). The implication of this first line of research is that semantic congruency facilitates attentional selection at the location of the congruent stimuli and boosts perceptual processing and performance. This attentional focusing is not directly linked to the stimuli *per se* and therefore cannot be classified as “bottom-up” or “stimulus-driven” (Corbetta and Shulman, 2002; Koelewijn et al., 2010; Talsma et al., 2010). Rather, it is enhanced by the semantic congruency of the stimuli. The facilitation is therefore due to learned semantic associations and as such must be classed as a “top-down” process. Yet in these studies, the attentional engagement is exogenously controlled via semantic priming, and voluntary, endogenous attention was not investigated.

Contrasting with the previous literature, a more recent second line of research investigating the same cognitive processes arrived at a different conclusion. A recent study found that task performance involving two cross-modal objects diminished in the presence of a third modality if that task-irrelevant object was semantically congruent with one, but not both, of the two task-relevant objects, especially when the task-relevant objects were themselves incongruent (Misselhorn et al., 2016). A similar effect was observed in two other studies. When participants attended to one of two laterally presented visual streams of letters while performing a sequential matching task, their response times (RT) were significantly longer, when incongruent, task-irrelevant letter sounds were presented as well. The increase in RTs on these trials coincided with increased fMRI activation in the anterior cingulate cortex and over fronto-central EEG sensors (Zimmer et al., 2010a, b). These findings suggest that semantically incongruent stimuli induce a cognitive conflict between the components of a multi-modal stimulus and subsequently, likely exogenously, recruit executive attentional resources to resolve the conflict, thus reducing the efficiency of multi-sensory integration of semantically congruent stimuli. Thus, this line of research suggested that the voluntary allocation of attentional resources in processing semantically congruent stimuli can be disrupted by endogenously unattended, task-irrelevant semantically incongruent stimuli.

These two lines of research imply different mechanisms for the interaction of semantic congruency and attentional selection. While the former suggests that congruent stimuli at an attended location boosts performance, the latter implies that incongruent and unattended stimuli recruit exogenous attention, and so divert resources from processing the congruent stimuli in the attentional focus, which reduces behavioral performance.

Here, we aimed to address these conflicting findings by investigating the interaction of attentional focus and semantic congruency in greater detail. We systematically varied the semantic congruency of three objects (a visual object, an auditory sound, and a visual background scene) in single- and cross-modal combinations, under different attentional foci and under conditions of explicit semantic congruence processing. Participants in three different groups directed their attention to two of three objects in the stimuli and made semantic congruency judgments for two attended stimuli. This allowed us to observe behavioral performance under conditions that replicated and extended critical features of the two lines of research yielding conflicting evidence. We were able to evaluate whether performance for attended congruent stimuli is increased or diminished in the presence of a distracting unattended and incongruent stimulus.

## Materials and Methods

### Participants

Participants (*n* = 87, mean age 25.53 years, SD 3.71, and 43 male) were recruited from the student population of the University Hamburg and participated for a small payment. They all had normal hearing and normal or corrected-to-normal vision. The study was approved by the ethics committee of the German Psychological Society (JG072015) and was conducted in accordance with the principles of the Declaration of Helsinki on human subject research.

### Experimental Design and Stimuli

To investigate the interaction of semantic congruence and attentional focus on the processing of multi-sensory stimuli, we created 3-object-component stimuli consisting of a visual object in the foreground (V), and typical auditory object (A) associated with the visual object (V), and a visual background scene (B). Semantic congruence was designed as a within-subject factor and varied between stimulus components, giving rise to the following 5 experimental conditions^1^ (see also Table 1): (1) none of the components are semantically congruent (coded as III), (2) V and A are congruent (coded as CCI, 1st and 2nd components are congruence), (3) V and B are congruent (coded as CIC, 1st, and 3rd component are congruent), (4) A and B are congruent (coded as ICC, 2nd and 3rd components are congruence, and (5) all components are congruent (coded as CCC).

**TABLE 1:** Experimental conditions and example objects comprising the cross-modal stimuli conditions.

**Condition code**	**Visual**	**Auditory**	**Background**
III	Door bell	Ambulance	Sky
CCI	Duck	Duck	Phone booth
CIC	Fire truck	Church bell	Burning house
ICC	Vacuum cleaner	Door bell	Door with bell
CCC	Lion	Roar	Savannah

*Condition codes are listed in the order visual-auditory-background. C indicates semantic congruency between the respective components, I indicates incongruency with the other components.*

Endogenous attention was manipulated as a between-subject factor in 3 groups: visual object and auditory sound (VA, *n* = 30, 16 males); visual object and background (VB, *n* = 27, 13 males); and auditory sound and background (AB, *n* = 30, 14 males). Participants in each group were instructed to focus their attention on the two object components of their group and judge these 2 components accordingly (see section “Experimental Task and Procedure” below).

**FIGURE 1:**
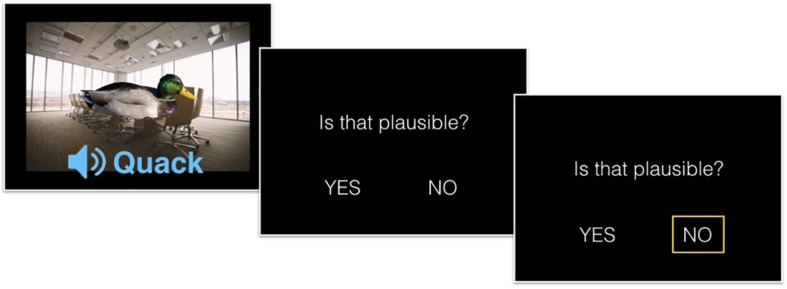
Experimental Task. Participants were presented with a 3-components stimulus compound consisting of a visual foreground object (V), an auditory sound (A) pertaining to the foreground object, and a visual background image (B). Each group of participants were instructed to focus their attention on 2 components and judge the semantic congruency of them (i.e., make plausibility judgment). In the example (presented to the AB group), the visual and auditory components are congruent, whereas the background is incongruent to the two foreground components. The displayed response (“not plausible”) is a correct response for participants in the AB group.

Visual objects were pictures of animals and everyday items, auditory objects were typical sounds of these visual objects, and background scenes depicted typical contexts in which the visual or auditory object could be found (see Figure 1 for an example). Incongruent combinations were created by randomly pairing an indoor object with an outdoor background (or sound) and vice versa. Upright pictures of the visual objects were scaled to a height of 250 pixels (px) [7.6 degrees of visual angle (dva)], horizontal pictures were scaled to a width of 510 px (16.13 dva, mean height 252.82 px, SD 52.41 px, mean width 315.48 px, and SD 99.98 px). The background pictures were scaled to 768 × 1024 px (25.36 × 33.4 dva) and presented with a gray frame on a Samsung SyncMaster 2443DW screen. The sounds were presented via headphones with a volume of ∼65 DB. All stimulus aspects were presented simultaneously with the foreground picture centered on the background (see Figure 1 for an example).

### Experimental Task and Procedure

After obtaining informed consent from the subject the experimenter instructed the participants about the goals of the study, the 3-component nature of the stimuli, and the attentional focus that they should maintain throughout the experiment. Participants were instructed to evaluate the congruency of the two components in their attentional focus, which was framed as a judgment of plausibility. Initial pilot data suggested that participants understood the term “plausible” better than “congruent.” In the main experiment it was explained to the participants that their plausibility judgment referred to the semantic congruence of the two components in question. They were told to respond as quickly and as accurately as possible with either the left and right arrow key representing a “YES” or a “NO” answer. The assignment of the response to the two response keys was counterbalanced across participants who responded with their index and middle finger of their dominant hand. Prior to the main experiment each participant completed a few training trials from the CCC and III condition until they responded correctly in 5 consecutive trials. In the main experiment, participants completed 150 trials (30 in each condition). Each stimulus was presented for a variable duration (depending on the duration of the sound clip (mean duration 1.53 s). Participants had to respond within 4 s. Failure to response in this window resulted in a missing trial. Trials were separated by 1.4 s. The experiment lasted around 15 min.

### Data Preprocessing

Response accuracy and RT were collected as experimental data. All missing trials were removed from the data. Outliers were defined as 2 SD above the mean of the square-root transformed RT data and also removed from the experimental data. Finally, the effect of stimulus duration was removed using a regression approach: RT data were log-transformed and regressed onto the stimulus duration (general linear model with stimulus duration and intercept as predictor variables). Duration-predicted RTs were removed by subtractions, and residuals from this regression were back projected into the original RT space and subjected to exponential transformation.

### Statistical Analysis

Response accuracy and RT were analyzed with linear mixed-effects models using the nlme package in R. Specifically, we set up omnibus mixed-effects repeated measures ANOVAs with the within-subject factor *Condition* (III, CCI, CIC, ICC, and CCC) and the between subject-factor *Focus* (VA, VB, and AB) using the formula:

DV∼conditionfocus*,random=∼1|id/condition

where DV is the dependent variable “percent error,” “RT (correct trials},” or “RT (incorrect trials).” *Post hoc* Bonferroni-adjusted contrasts were carried out using the multcomp package in R.

### Cognitive Computational Modeling

Hierarchical Bayesian parameter estimation with the drift diffusion model (DDM) yielded group and individual participant estimates the drift rate (v), the boundary separation (a), and the non-accumulation time (t), also called the non-decision time (Ratcliff and McKoon, 2008). The DDM treats a binary decision as the result of an evidence accumulation process, in which the gathering of evidence for one or the other option is modeled as a Gaussian random walk that drifts at a certain rate toward one of two decision boundaries representing the two alternative options [see Ratcliff and McKoon (2008) for a schematic of the model]. Once one of these boundaries is crossed, a decision for this option is made. There are 4 primary free parameters in the DDM, whose optimized values are determined during model fitting: (1) the drift rate *v* governs the speed of evidence accumulation, corresponds to the slope of the random walk, and reflects choice difficulty, (2) the boundary separation *a* represents the distance between both decision boundaries and models how cautious a decision maker is with higher caution corresponding to a larger boundary separation, (3) the starting point *z* is the point between both decision boundaries at which the evidence accumulation starts. Although this parameter is unused in this study (i.e., is set to a/2) it can model general biases toward one or the other option, (4) the non-decision time *t* captures all aspects of the RT that are not related to evidence accumulation, i.e., stimulus-encoding, feature selection, action-planning, and action-execution time.

Model fitting with the HDDM package in Python (Wiecki et al., 2013) offered a Hierarchical Bayesian workflow using Markov Chain Monte Carlo (MCMC) techniques. In most cases this yields more stable results than traditional Maximum Likelihood estimation and includes measures of estimation uncertainty in the form of posterior distributions of parameters. In addition, subject-specific parameter values are sampled from an overarching group distribution, which is updated using the data from all participants. This usually leads to more stable optimized parameter solutions, while also allowing for individual variability in these estimates.

The package offers a model parameterization depending on the experimental factors, e.g., one could model different drift rates for all conditions or for all groups or any combination of them. We compared these different model variants using the deviance information criterion (DIC), a model comparison index similar to the Bayesian information criterion (BIC), but applicable for Bayesian analysis using MCMC sampling. A difference in DIC scores of 15 and above is considered meaningful (Spiegelhalter et al., 2002).

Each model variant was fit using the HDDM package (Wiecki et al., 2013) with 4 chains and 7000 samples following a burn-in phase of 500 samples to reduce the dependencies on initial values and to reach a steady state of the chain. Convergence was tested through visual inspection of the chains and by calculating the R̂ statistic (Gelman and Rubin, 1992), which compares within-chain and between-chain variance. The threshold for non-convergence was set at 1.05. We used the HDDM defaults as group-level priors, namely the drift rate was modeled as a group-level normal distribution [N (μ,σ^2^)], whose parameters μ_*v*_ and σ_*v*_^2^ were modeled as N (2,3) and half-normal distribution HN (2) (2 being the variance parameter). The boundary separation was modeled as a Gamma (G) distribution, whose parameters μ_*a*_ and σ_*a*_^2^ were modeled as G (1.5,0.75) and HN (0.1) distributions. Finally, the non-decision time was also modeled as a normal distribution, whose parameters μ_*t*_ and σ_*t*_^2^ were modeled as N (2,3) and HN (1) distributions. We compared parameter estimates for the different levels of each factor by mean of the group posterior distribution.

## Results

### Analysis of Errors and Response Times

**FIGURE 2:**
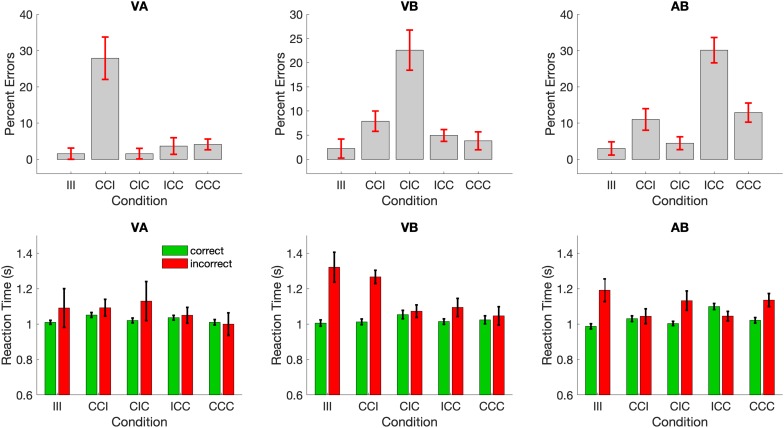
Mean percent errors and response times. Participants in all 3 attention groups committed substantially more errors when the unattended stimulus component was incongruent to the other (congruent) components in the attentional focus.

We first inspected the percent errors in all 3 groups of subjects with different attentional foci across all 5 stimulus conditions. To be counted as an error, the participant would have to (a) respond “not plausible” to two congruent components in the attentional focus (e.g., in group VA visual: lion, auditory: roar, background: swimming pool) or (b) respond “plausible” to two incongruent components in the attentional focus (e.g., in group VA visual: fire truck, auditory: church bell, background: burning house). Overall, participants only made few errors on the task (overall percentage of errors: (group VA: 8.1% incorrect, 91.2% correct, 0.7% missing trials, group VB: 9.1% incorrect, 90.5% correct, 0.4% missing trials, group AB: 13.2% incorrect, 86.3% correct, and 0.5% missing trials). However, despite the overall low number of errors the different groups made substantially more errors in different, yet specific conditions in the task (see Figure 2): whenever the unattended component was incongruent to the two congruent components in the attentional focus (i.e., in group VA – CCI, in group VB – CIC, in group AB – ICC), the error rate was substantially higher, than in all other conditions.

A mixed effects ANOVA with the within-subject factor *Condition* and between-subject factor *Focus* confirmed a significant main effect of *Condition* (*F*_4,336_ = 10.48, *p* < 0.0001) and a significant *Condition* × *Focus* interaction effect (*F*_8,336_ = 14.42, *p* < 0.0001). Subsequent, Bonferroni-adjusted contrasts between the different stimulus conditions revealed that the interaction effect was driven in each group by a significant difference between the critical condition (in group VA – CCI, in group VB – CIC, in group AB – ICC) and all conditions (all *z*-values > 3.9, *p* < 0.001).

**FIGURE 3:**
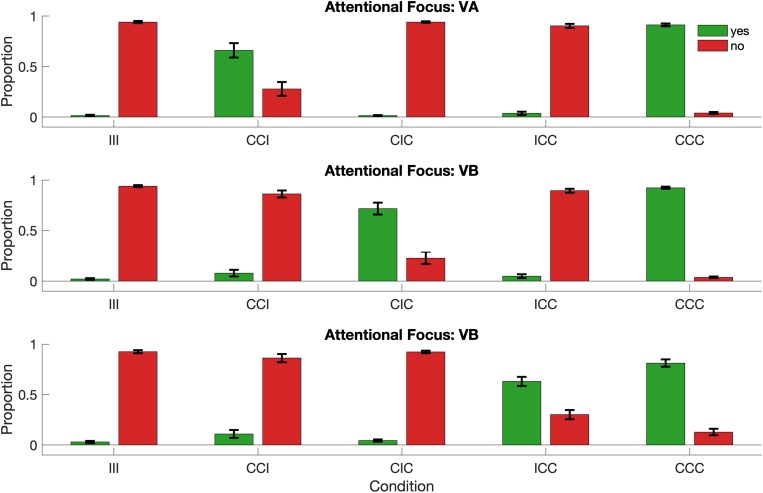
Response bias in the data (proportion scale). Data are displayed according to the actual response of the participant (“YES” and “NO”) for all conditions in all groups. While in some conditions subject responded with “NO” more frequently, there was no overall evidence for a general response bias in the data.

Across all conditions a “NO” response (not plausible) was more frequently correct (for instance in conditions III, CIC, and ICC for the VA group) than a “YES” response (namely in condition CCI and CCC for the group VA). Thus, it is conceivable that participants learned about this subtle response bias and that they committed more errors in the critical conditions. The possibility of such a response bias is detectable, if the data are sorted according to the response itself instead of the response accuracy. If a response bias was present in the data, we would expect to see higher frequency of “NO” response across all conditions in all groups. Figure 3 demonstrates that this is not the case. In fact, the pattern found in this analysis mirrors the finding from Figure 2: in the critical conditions there were a significant number of “NO” responses (i.e., and incorrect decision), whereas in the non-critical condition there were mostly “NO” and “YES” responses (correct responses depending on the condition). Importantly, this figure reveals that there was no overall bias toward “NO” responses.

In contrast, analyses of the RTs did not yield an equally systematic pattern of findings despite a significant effect for *Condition* (*F*_4,331_ = 7.11, *p* < 0.001) and for *Condition* x *Focus* (*F*_8,331_ = 5.53, *p* < 0.0001) for RT in *correct trials* and a significant effect for *Condition* (*F*_4,170_ = 2.78, *p* = 0.029) and a trend-level *Condition × Focus* interaction (*F*_8,170_ = 1.86, *p* = 0.069) for RT in *error trials*. Subsequent Bonferroni-adjusted *post hoc* contrasts revealed that for RTs in *correct trials* only, condition ICC in group AB was significantly longer than all other conditions (all *z*-values > 4.1, *p* < 0.001). In addition, for RTs in error trials, conditions III, and CCI in group VB were significantly larger than all other conditions (all *z*-values > 3.16, *p* < 0.05). There were no additional RT effects in any of the other groups.

## Conclusion

In conclusion, participants made significantly more errors whenever the unattended stimulus was semantically incongruent with the two congruent stimuli in the focus of endogenous attention. However, a corresponding increase in RT on those error trials could not be found.

### Computational Cognitive Modeling

In the next step we applied cognitive computational modeling to these data to gain additional insights into the cognitive processes governing the responses in this task. The drift diffusion model (DDM) (Ratcliff and McKoon, 2008) is particularly well-suited for modeling the decisions in this task. A decision in the DDM is the result of an evidence accumulation process, which “drifts” at a specific rate to one of two decision boundaries representing the two decision options. In our case we defined the two options as “correct” and “incorrect” responses as this form of data coding has provided fruitful insights into the speed-accuracy trade-off present in most behavioral decision-making paradigms (Ratcliff and Rouder, 1998; Steinweg and Mast, 2017).

**FIGURE 4:**
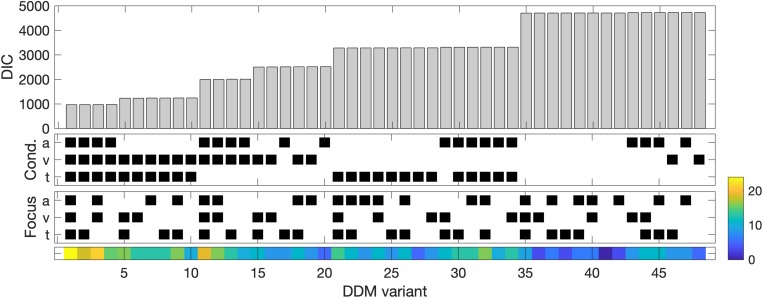
Model comparison. **Top**, deviance information criterion (DIC) for all model variants tested in this study sorted by size. DIC balances model fit (deviance, difference between fitted model and data), and model complexity (number of free parameters). **Middle**, indicator variable for the model variant. A black dot for a particular model variant indicates that the parameter listed in the row is modeled separately for each level of the factors Condition (5 levels) or Focus (3 levels corresponding to the three experimental groups). **Bottom**, number of parameters (color-coded) for each model variant indicating model complexity.

We compare variants of the DDM with different configurations of free parameters. Each of the 3 selected parameters (drift rate *v*, boundary separation *a*, and non-decision time *t*) could be modeled as a single parameter across all stimulus conditions or as a single parameter across all groups. In contrast, each parameter could be also modeled separately for each stimulus condition and for each attention group. We systematically compared all possible variants of the DDM using their DIC score (see Figure 4).

**FIGURE 5:**
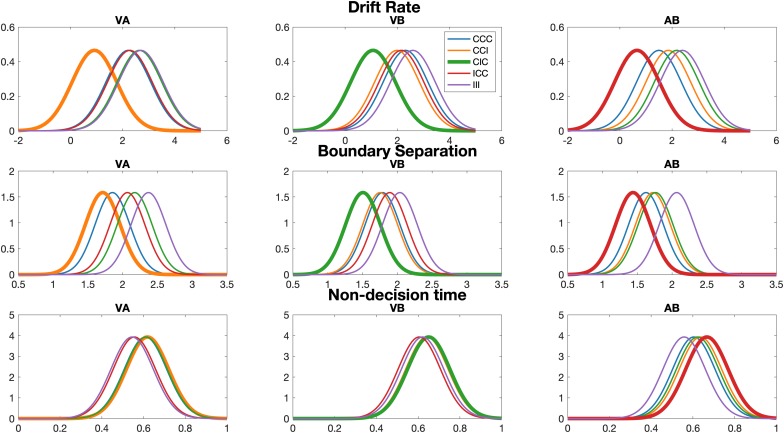
Group posterior distribution for all parameters in each condition and attention group. The critical conditions are plotted with thick lines. Note that the variance parameter for the normal distribution shown here is always the same for a specific parameter (i.e., each condition in each group has the same variance parameter).

This model comparison analysis reveals that the model variants in which all three parameters are modeled separately for each condition provided the best model fit, but there are no meaningful differences between these regarding the group impact (DIC differences < 15). Nevertheless, model 1 (the model with the lowest DIC score) also provided separate parameter distributions for each group, which allowed us to compare parameter distribution for each condition in each group. Given that the critical condition corresponded to different stimulus configurations in each group, model 1 thus provides the granularity to detect the effect of critical conditions in the parameter distributions. We show the group posterior distributions for all parameters in Figure 5.

Interestingly, the drift rate parameter (Figure 5, top) for the critical condition is always smaller than all other conditions in each group. This resembles the patterns of errors seen in the behavioral analysis above: whenever the unattended stimulus component was incongruent to the two other components in the attentional focus, we observed a reduced drift rate parameter. Similarly, the boundary separation parameter for the critical condition is also the smallest compared with all other conditions, but this pattern is less clear than for the drift rate. Finally, no such pattern of the critical conditions was observed for the non-decision time.

**FIGURE 6:**
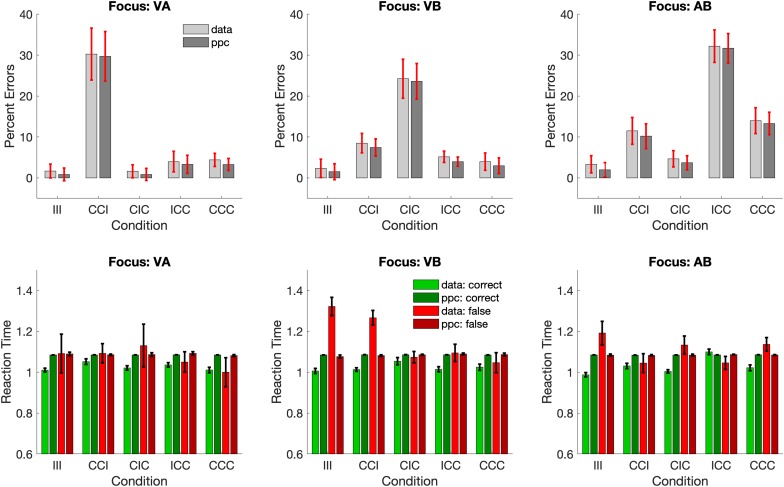
Posterior predictive checks. The simulated new data from Model 4 shows great accuracy in reproducing the choices **(top)**, but it does not capture the slight differences in the RTs in correct, and incorrect trials **(bottom)**.

Having selected the best-fitting model from within a family of model variants does not insure that the model actually fits the data. This can be tested using posterior predictive checks (PPC), in which the model generates new data using the fitted parameters. These data are then compared to the original data. Below, we show the PPC findings for our selected Model 4, which simulated 500 new data points for the same number of subjects in each attention group. The response accuracy and correct and error RTs were then compared to the original data (see Figure 2). Figure 6 shows the findings from this posterior predictive check.

The correct and incorrect responses of the PPC match the original data with high accuracy. However, the simulated RTs do not fit with the subtle differences in correct and incorrect RTs in the data. In fact, it seems that in the PCC simulations all conditions in all groups are modeled with essentially the same mean RT.

## Discussion

We found a specific effect of attentional focus on the processing of our multi-sensory stimuli. Whenever the unattended stimulus is incongruent with the two others in the attentional focus, participants made significantly more errors in semantic congruency judgments than in any other stimulus condition. This effect is paralleled by a significantly reduced drift rate parameter in these stimulus conditions as revealed in our drift diffusion modeling. RTs do not show a similar increase in RTs in error trials in these specific conditions. Rather, the pattern in error RTs seems to be driven by non-systematic increases in specific stimulus conditions, but unrelated to the attentional manipulation.

Our findings are in line with those studies demonstrating that semantically incongruent stimuli outside the focus of attention can capture those processing resources and disrupt the processing and evaluation of the attended stimuli (Zimmer et al., 2010a, b; Misselhorn et al., 2016). Indeed, it seems that in our data semantically incongruent stimuli induce a re-focusing of attention, such that the incongruency of the unattended stimulus is then considered leading to an incorrect (incongruent) judgment. If this was the case, then from the perspective of the participants, they would be making correct responses. This could be the reason why the RTs between correct and incorrect trials in these critical conditions are almost identical (see Figure 2 bottom, VA – CCI, VB – CIC, AB – ICC). Such an account would still be consistent with the neuroimaging findings from earlier studies demonstrating higher activations in anterior cingulate cortex (ACC) implying a processing of the conflict between semantically incongruent stimuli (Zimmer et al., 2010b). Other previous studies that also investigated sematic congruency in a multimodal context also observed higher ACC activations during the processing on incongruent stimuli (Weissman et al., 2004) reminiscent of the findings on conflict detection in the Stroop task (Fan et al., 2003). The brains in our subjects could be detecting the incongruency between the one of the previously attended congruent stimuli and the incongruent previously unattended, but now re-focused stimuli and yet still make an incongruent (but from their perspective correct) judgment. Of note, our primary finding of attentional capture of semantically incongruent stimuli occurs irrespective of the modality of the stimuli suggesting that we observed a general effect between attentional selection that is influence by sematic (in) congruency.

Nevertheless, previous studies investigating semantic congruency with multi-modal stimuli also observed modulation of brain activity in the primary uni-sensory areas. In general, activation in primary sensory cortices in boosted if the modality is task-relevant (Weissman et al., 2004) and (semantically) congruent with other modality in stimulus compound (van Atteveldt et al., 2004), although an active encoding task might alleviate the advantage for congruent stimulus compounds (van Atteveldt et al., 2007). In fact, other studies have also reported increased activity in higher activations for incongruent stimuli in primary sensory areas of the target modality (Weissman et al., 2004). These neural findings generally support the influence of endogenous and exogenous attention on the processing of multi-modal semantic congruency: as attention is directed toward a target modality (endogenous attention) the activation in those primary uni-sensory is increased, but if the target modality is incongruent with an unattended modality, processing resources are also recruited (exogenous attention) and activation in related brain regions is also increased. Our findings support the exogenous attention recruitment hypothesis: that is, participants committed significantly more errors whenever the unattended stimulus was incongruent to the two stimuli in the attentional focus, irrespective of the sensory modality of the stimuli. This points toward a general attentional bias for the processing of semantic incongruency.

In our study, the differences between the critical conditions mentioned above and the other conditions involving one incongruent stimulus is that in the critical conditions, the participants are initially primed to process a congruent stimulus combination because it is in the attentional focus. The incongruent stimulus then captures attentional resources leading to a refocusing of attention and prompting the participants to make more “incongruent” judgments, which are counted as “incorrect” here from the standpoint of an all-knowing observer, who knows what the participant should focus on. That is, in the critical condition the congruent stimulus pair comes first, whereas in the other conditions (e.g., for VA – CIC and ICC) the attention is already focused on an incongruent stimulus pair, which is in most cases correctly detected through an “incongruent” judgment. This could be a potential reason for the lack of a systematic response time difference between the conditions: participants make the identical “incongruent” judgment, which could take approximately the same amount of processing time, but in the critical condition these responses are counted as incorrect.

Our drift diffusion modeling revealed that the observed increases in error rate in the critical conditions involving an unattended incongruent stimulus were paralleled by a significantly lower drift rate (Figure 5). The drift rate in diffusion models describes the speed of evidence accumulation until a decision is reached, when the diffusion process hits one of the two decision boundaries (Ratcliff and McKoon, 2008). In terms of cognitive processing, a lower drift rate in the presence of constant boundary separation means that participants take longer to accumulate evidence over the same boundary. This is commonly an indicator of difficulty induced by task condition or some other variable. The situation in our critical conditions would qualify as increased difficulty of evidence accumulation if exogenous attention engaged by incongruent, task-irrelevant stimuli interfered with endogenous attention. This implication has further evidence in that our model fitting of the DDM resulted in a significantly reduced boundary separation parameter in the critical condition, meaning that the representation of the two task options of congruent vs. incongruent was less stably separate, likely due to interference from exogenous attention to the incongruent distractor. The combination of lower drift diffusion rates and reduced boundary separation is consistent with our observation that RTs were not reduced in the critical conditions, and with the increased error rates in decisions in the critical conditions. Thus, the cognitive computational modeling revealed cognitive dynamics that a more conventional analysis of RTs would have missed.

Our RT data (Figure 2) also revealed a small number of significant RT differences between correct and incorrect trials in some conditions in the VB and AB groups. However, there appears to be no systematic pattern in these differences that can be related to the experimental manipulation. A potential reason for these non-systematic effects could be that the overall error rate in the experiment is quite low leaving only a few error trials for computing an average error RT. It is therefore likely that some of these high error RTs are driven by outlying data points that were not detected in our preprocessing steps.

The low number and unsystematic occurrence of error trials is also the likely reason that the classic DDM failed to replicate the observed differences in RT in the posterior predictive check (Figure 6), while at the same time reproducing the pattern of errors quite accurately. In fact, the synthetic data generated from the fitted parameters of the classic DDM exhibited no difference in mean RTs for any condition in any group, which could be interpreted that the observed RT differences are unsystematic and cannot be accurately modeled by the classic DDM. One way of accounting for different RT distributions of correct and incorrect responses is to add parameters that model inter-trial variability of drift rate, starting point and non-decision time. We did not include these parameters, because our main interest was on the core DDM parameters such as drift rate and boundary separation, and estimating the latter can be compromised by adding the former (Boehm et al., 2018). However, this does not mean that the classic DDM is not suitable for modeling the data in our experiment. In fact, by tuning drift rate, boundary separation and non-decision time independently for each condition, the model is capable of reproducing the pattern of correct, and incorrect responses with a high degree of accuracy (Figure 6). This reinforces the interpretation from above that a lower drift rate in the critical condition indicates an increased processing demand due to the refocusing of the attentional focus to include the (formerly) unattended, incongruent stimulus.

Semantic congruency is a powerful amplifier of multi-sensory integration leading to higher brain activation (Doehrmann and Naumer, 2008) and better performance (Taylor et al., 2006; Steinweg and Mast, 2017). In addition, it can focus non-voluntary, “stimulus-driven” attention toward congruent stimuli and can boost perceptual processing resources at their location (Iordanescu et al., 2008, 2010). Moreover, semantic incongruency can disrupt the perceptual processing of stimuli in the attentional focus (Zimmer et al., 2010a, b). The findings of the present study are in line with these previous findings as we were able to show that semantic incongruency – independent of the stimulus modality – led to a re-focusing of attention to include the previous unattended (and incongruent) stimulus. It thus seems that our perceptual system is finely attuned to detect semantic incongruencies, even at a pre-attentive state. From a predictive coding perspective (Rao and Ballard, 1999; Friston, 2005), such incongruencies constitute prediction errors (violations of our expectations), which prompts the reallocation of processing resources via exogenous attention in implicitly attempting to resolve the incongruency of the percept. This would imply that the behavioral performance of our subjects in the critical condition is not erroneous, but rather adaptive to the needs for further cognitive processing independently of modality.

## Data Availability

The datasets generated for this study are available on request to the corresponding author.

## Ethics Statement

The studies involving human participants were reviewed and approved by the German Psychological Society. The patients/participants provided their written informed consent to participate in this study.

## Author Contributions

JG designed the research. JS-L collected the data. JG, JS-L, and MS analyzed the data. SG helped with computational modeling. All authors wrote the manuscript.

## Conflict of Interest Statement

The authors declare that the research was conducted in the absence of any commercial or financial relationships that could be construed as a potential conflict of interest.

## Footnotes

1 The three letters in the condition codes always refer to 1, visual; 2, auditory; 3, background in that order.
